# Health Care Transition Experiences of Males with Childhood-onset Duchenne and Becker Muscular Dystrophy: Findings from the Muscular Dystrophy Surveillance Tracking and Research Network (MD STARnet) Health Care Transitions and Other Life Experiences Survey

**DOI:** 10.1371/currents.md.7de8a1c6798d7a48d38ea09bd624e1cd

**Published:** 2018-08-21

**Authors:** Pangaja Paramsothy, Adrienne R. Herron, Molly M. Lamb, Kathi Kinnett, Jodi Wolff, Michele L. Yang, Joyce Oleszek, Shree Pandya, Annie Kennedy, Darryl Cooney, Deborah Fox, Daniel Sheehan

**Affiliations:** National Center on Birth Defects and Developmental Disabilities, Centers for Disease Control and Prevention, Atlanta, Georgia, United States of America; National Center for HIV/AIDS, Viral Hepatitis, STD and TB Prevention, Centers for Disease Control and Prevention, Atlanta, Georgia, United States of America; Department of Epidemiology, Colorado School of Public Health, Aurora, Colorado, United States of America; Parent Project Muscular Dystrophy, Hackensack, New Jersey, United States of America; Santhera Pharmaceuticals Inc., Burlington, Massachusetts, United States of America; Department of Pediatrics, Children's Hospital Colorado, Aurora, Colorado, United States of America; Department of Neurology, University of Rochester, Rochester, New York, USA; Parent Project Muscular Dystrophy, Hackensack, New Jersey, United States of America; Division of Statistics and Data Science, RTI International, Research Triangle Park, North Carolina, United States of America; Department of Malformations Registry, New York State Department of Health, Albany, New York, USA; Department of Pediatrics, Jacobs School of Medicine and Biomedical Sciences, University at Buffalo, The State University of New York, Buffalo, New York, United States of America

## Abstract

**Introduction:**

As the proportion of males with Duchenne muscular dystrophy (DMD) surviving into adulthood increases, more information is needed regarding their health care transition planning, an essential process for adolescents and young adults with DMD. The objective of this study was to describe the health care transition experiences of a population of males living with Duchenne or Becker muscular dystrophy (DBMD).

**Methods:**

The eligible participants, identified through the Muscular Dystrophy Surveillance Tracking and Research Network (MD STARnet) surveillance project, were 16–31 years old and lived in Arizona, Colorado, Georgia, Iowa, or western New York (n=258). The MD STARnet Health Care Transitions and Other Life Experiences Survey was conducted in 2013 and administered online or in a telephone interview. Sixty-five males (25%) completed the survey. Among non-ambulatory males, response differences were compared by age group. Statistical comparisons were conducted using Fisher’s exact test, or when appropriate, the Chisquare test.

**Results:**

Twenty-one percent of non-ambulatory males aged 16–18 years, 28% of non-ambulatory males aged 19–23 years, 25% of non-ambulatory males aged 24–30 years, and 18 ambulatory males had a written transition plan. Nineteen percent of non-ambulatory males aged 24–30 years had delayed or gone without needed health care in the past 12 months. Among non-ambulatory males aged 24–30 years, 75% had cardiology providers and 69% had pulmonology providers involved in their care in the past 12 months. Twentyeight percent of non-ambulatory males aged 19–23 years and 25% of non-ambulatory males aged 24–30 years reported that they did not receive health care or other services at least once because they were unable to leave their home. Non-ambulatory males aged 16–18 years (29%) were less likely to have ever discussed how to obtain or keep health insurance as they get older compared to non-ambulatory males aged 24-30 years (69%) (p <0.01).

**Discussion:**

This study identified potential barriers to the successful health care transition of males with DBMD. The results of this study may indicate a lack of targeted informational resources and education focused on supporting the transition of young men with DBMD as they age from adolescence into adulthood within the healthcare system. Future studies could determine the reasons for the potential barriers to health care and identify the optimal transition programs for males with DBMD. There are a few online resources on transition available to adolescents and young adults with special health care needs.

## **Introduction**

As a typical part of healthy development, individuals experience many different types of transitions across their lifespan. The transition from adolescence to adulthood is a process involving a variety of aspects and the health care transition is but one component. A successful health care transition should be a purposeful and systematic movement from a pediatric to an adult heath care system.^1^^, ^2 In recent years, emphasis has been placed on the health care transition experiences of youth with special health care needs. One of the objectives of Healthy People 2020 is to increase the percentage of youth with special health care needs whose health care provider has discussed transition planning with them.^3^

Duchenne muscular dystrophy (DMD) and Becker muscular dystrophy (BMD) are neuromuscular disorders caused by a mutation in the dystrophin gene on the X-chromosome, which leads to progressive muscle weakness.^4^^, ^5 Individuals diagnosed with DMD typically lose the ability to walk independently by age 13 years, and progress to cardiac and respiratory complications.^4^^, ^5 BMD is typically a milder phenotype with a variable progression.^5^^, ^6 In the recently published revisions to the Duchenne muscular dystrophy (DMD) care considerations, health care transition planning is emphasized.^7^

Positive health care transition experiences have been associated with better health outcomes. Satisfaction with transition of care has been associated with better social and emotional quality of life among adolescents with neuromuscular disorders.^8^ Healthcare transition programs have been associated with better disorder-specific health outcomes in young adults with diabetes^9^^, ^10 and those who have undergone renal transplantation^11^^, ^12.

The survival of individuals living with DMD has increased, with the median age of survival now in the mid-twenties^13^^, ^14^, ^15 and many individuals living into their third decade. It is essential for all adolescents living with Duchenne or Becker muscular dystrophy (DBMD) to have health care transition planning. In June of 2011, Parent Project Muscular Dystrophy, a nonprofit family-advocacy organization, convened an expert panel to discuss how to improve the transition experiences of young men with DMD.^16^ The major goal that emerged from this meeting was the need to change the perception that DMD is only a disease of childhood. A few qualitative studies have examined the transition experiences of young men with DMD living in England^17^^, ^18^, ^19, Canada^20^^, ^21^, ^22 and Japan^23^, however little information is available on the transition experience of adolescent and young men living with DMD in the United States. The objective of this manuscript is to describe the health care transition experiences of young males living with DBMD in certain areas of the United States who participated in the MD STARnet Health Care Transitions and Other Life Experiences Survey.

## 

## **Methods**

Starting in 2002, the Centers for Disease Control and Prevention funded a population-based surveillance system to determine the prevalence of childhood-onset DBMD and to collect information on clinical practices and health outcomes. An in-depth description of the MD STARnet methodology has been published.^24^ Beginning in 2004, MD STARnet retrospectively identified and longitudinally followed all individuals diagnosed with DBMD, who were born since January 1, 1982, who were symptomatic by age 21, and who resided in four US sites: Arizona, Colorado, Iowa, and 12 counties in western New York State. Georgia was added to the surveillance program in 2005 and Hawaii in 2008. Medical record data were abstracted from neuromuscular clinics, emergency departments, hospitals, and vital records. Annual medical record abstraction was conducted through December of 2011. For cases identified from September 2011 through December 2011, record abstraction was conducted through 2012.

A committee of clinical experts reviewed abstracted diagnostic data to assign each individual a case definition (definite, probable, possible, asymptomatic, affected female, or not DBMD).^25^ At the end of data collection, another group of experts reviewed mutation information from genetic testing, western-blot dystrophin levels from muscle biopsy, age when ambulation first ceased, steroid treatment, and age at onset of DBMD symptoms in order to classify males as Duchenne phenotype, Becker phenotype, or unable to determine.^26^

In 2013, from April through October, MD STARnet conducted a survey among males with DBMD in order to describe their transition experiences in health care, living arrangements, education and employment, and quality of life. The health care transition sections of the survey consisted of questions that assessed mobility and respiratory function, health care providers and access to health care facilities, health care utilization, health care coordination and management, health insurance, and barriers to care. A working group consisting of MD STARnet clinicians, researchers, and representatives from advocacy organizations developed the survey questions. Some survey questions regarding the health care transition process were taken from two national surveys: the National Survey of Children with Special Health Care Needs (NS-CSHCN)^27^^, ^28 and the Survey of Adult Transition and Health (SATH)^29^. Questions were also based on the 2012 version of the MDA transition survey^30^ and a survey of adolescents and young adults living with spina bifida^31^. Questions were piloted at neuromuscular clinics in Iowa and western New York among non-resident males with DBMD who were 16 years or older.

Eligibility criteria for the MD STARnet transition survey included being identified in the MD STARnet system by December 2011, living in the surveillance area at the time the survey was conducted, being at least 16 years old at the time of the survey, having an MD STARnet case definition of definite or probable, and being the oldest living male affected with DBMD in the household. The survey was not conducted in Hawaii because of incomplete case ascertainment. The survey was available in English and in Spanish. The transition survey was internet-based and was administered using the Qualtrics system housed at the University of Iowa

**Ethical statement**

In Colorado, Georgia, Iowa and western New York, public health authority permitted medical record abstraction for DBMD. In Arizona, institutional review board (IRB) approval was obtained from the University of Arizona, and when needed, from other health care facilities where data collection occurred.

IRB approval for the MD STARnet transition survey protocol was obtained at each site. Site differences in survey methodology were due to differences in site IRB requirements. Individuals who were eligible for the survey were sent a letter inviting them to participate. For males under age 18 years in Georgia, the letter was addressed to a parent or legal guardian and asked permission to allow the eligible male to participate. Invitations included an online survey link and login information. Participants were also given the option to request a telephone interview, by filling out their contact information and returning it in the postage-paid return envelope. A study coordinator then contacted the participant. Online participants (Arizona, Colorado, and Georgia) reviewed a consent page before completing the questionnaire; phone participants issued a verbal consent after a staff member read the online consent form verbatim. Implied consent, as indicated by completing the survey, was approved for the Iowa site.

**Statistical analysis**

Two analysts conducted statistical analyses independently at different sites using SAS versions 9.3 and 9.4 (Cary, North Carolina). Investigators used medical record data abstracted through December 2011 to compare eligible males who completed the survey to eligible males who did not complete the survey. Transition experiences were expected to differ not only by age, but also by ambulation status. Therefore, analysts grouped participants into one of two categories, those who used a wheelchair full-time (non-ambulatory) and those who either used a wheelchair part-time or who were fully ambulatory, according to their responses to the survey question on current mobility. One male who responded “does not apply” to the mobility question was included in the non-ambulatory group. Responses among the non-ambulatory group were compared for three different age groups: 16–18, 19–23, and 24–30 years. Given the small sample size, responses for the ambulatory group were not compared by age. Statistical comparisons were conducted using Fisher’s exact test, or when appropriate, the Chi-square test.

## **Results**

A total of 258 males affected with DBMD were eligible for the transition survey and 65 of them completed the survey (completion rate was 25%). Among sites, Iowa had the highest percentage of males who completed the survey (Table 1). Completion rate also differed by race/ethnicity; Hispanics and Non-Hispanic Blacks were significantly less likely to complete the survey. Completion rates did not differ significantly by age, Duchenne or Becker phenotype, use of respiratory devices by the end of 2011, or ambulation status at the end of 2011.

**Figure:**
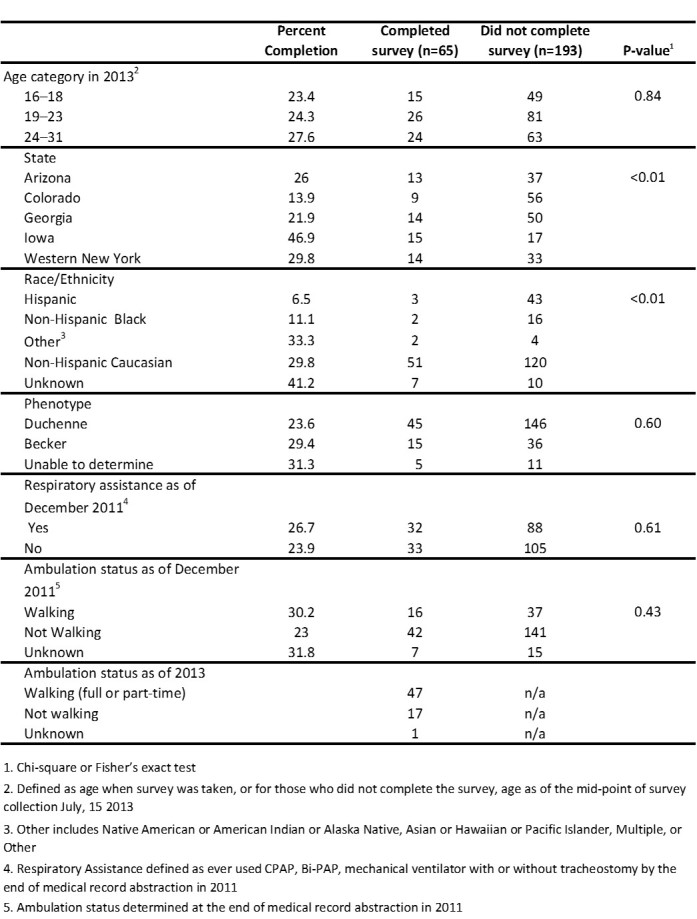
**Table 1.** Completion percent by study characteristics for the 258 males affected with Duchenne/Becker muscular dystrophy, aged 16–31 years, who were eligible for the 2013 MD STARnet Health Care Transitions and Other Life Experiences Survey

Among the 65 survey respondents, in 2013, 47 (72%) males indicated full-time current use of a wheelchair or scooter, 4 (6%) males indicated part-time use of a wheelchair or scooter, 13 (20%) indicated they walk without help. One male (2%) responded that the question does not apply and was added to the non-ambulatory group. Of the 48 males who were non-ambulatory, 43 (90%) were classified as Duchenne phenotype, one (2%) was classified as Becker phenotype, and for 4 (8%), phenotype could not be determined. Of the 17 males who were ambulatory, 14 (82%) were classified as Becker phenotype, 2 (12%) were classified as Duchenne phenotype, and for 1 (6%), phenotype could not be determined.

Table 2 displays results from questions regarding breathing and arm/hand ability. A higher percentage of non-ambulatory males aged 24-30 years (62%) used a ventilator with tracheostomy than males aged 19-23 years (6%) or 16-18 years (7%). While 44% non-ambulatory males aged 24-30 years reported that they could not use their hands, all of the non-ambulatory males aged 16-18 years and 19-23 years could use their hands.

**Figure:**
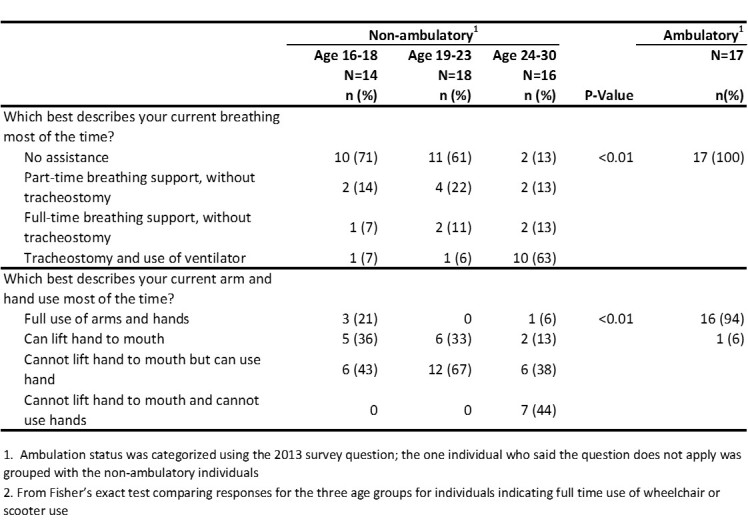
**Table 2.** Breathing, arm, and hand abilities, by age and ambulation status, 2013 MD STARnet Health Care Transitions and Other Life Experiences Survey of males affected with Duchenne/Becker muscular dystrophy, aged 16-30 years

Table 3 displays results from questions on health care access and health care provider types. . A high percentage of males (79-84%) reported having a health care provider to call when they had a question, having a place to go when they are sick (63-79%), and having a place that they go for routine preventative care (56-88%). However, a low percentage of all male respondents (14-31%) had access to a satellite or outreach clinic. Seventy-five percent of non-ambulatory males aged 24-30 years had cardiology providers involved in their care in the last 12 months and 69% of non-ambulatory males age 24-30 years had pulmonology providers involved in their care in the last 12 months.

**Figure:**
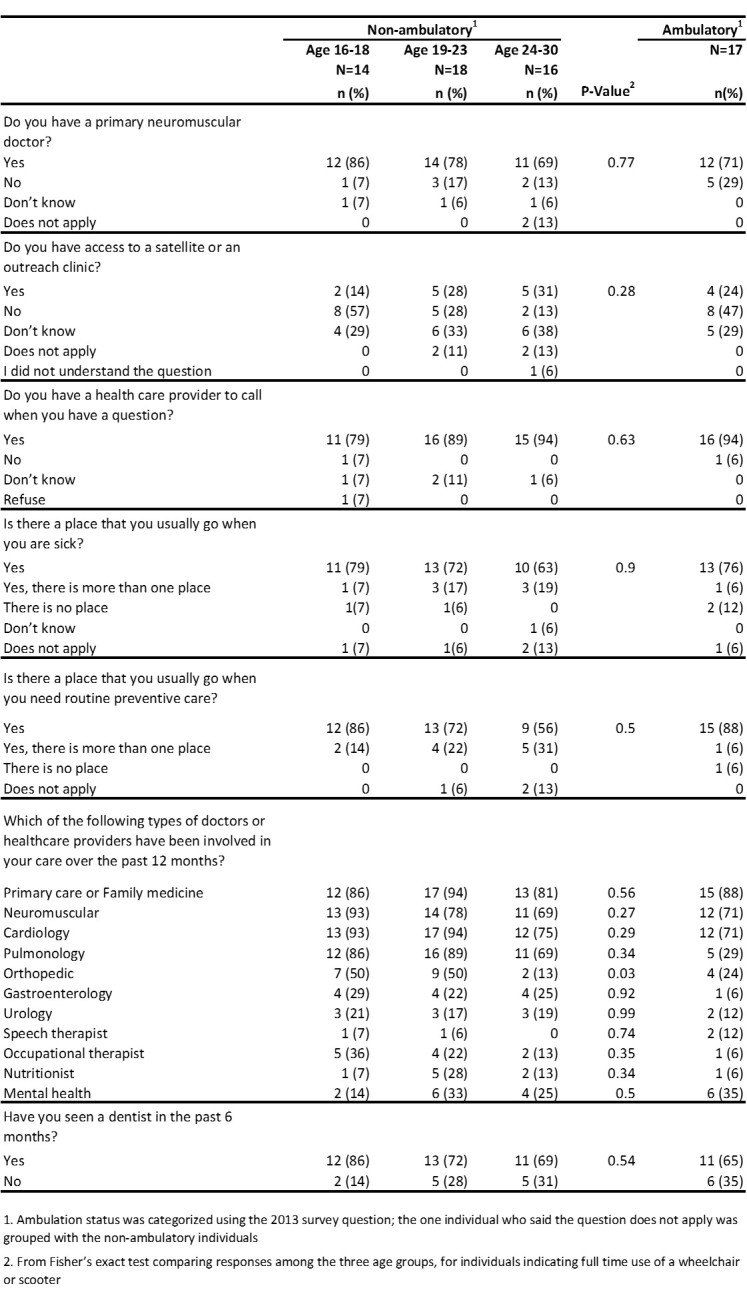
**Table 3.** Health care access and provider types by age and ambulatory status, 2013 MD STARnet Health Care Transitions and Other Life Experiences Survey of males affected with Duchenne/Becker muscular dystrophy, aged 16-30 years

Table 4 displays results from questions on the type of patients a provider treats and health care provider changes. Non-ambulatory adolescents were more likely to see a primary care/family medicine specialist, cardiologist, or pulmonologist who sees children only, while non-ambulatory males aged 24-30 years were more likely to see these specialist who saw adults only. A high percentage of non-ambulatory males aged 24-30 years (69%) and age 19-23 years (67%) reported ever changing a doctor or health care provider because the doctor was only treating children.

**Figure:**
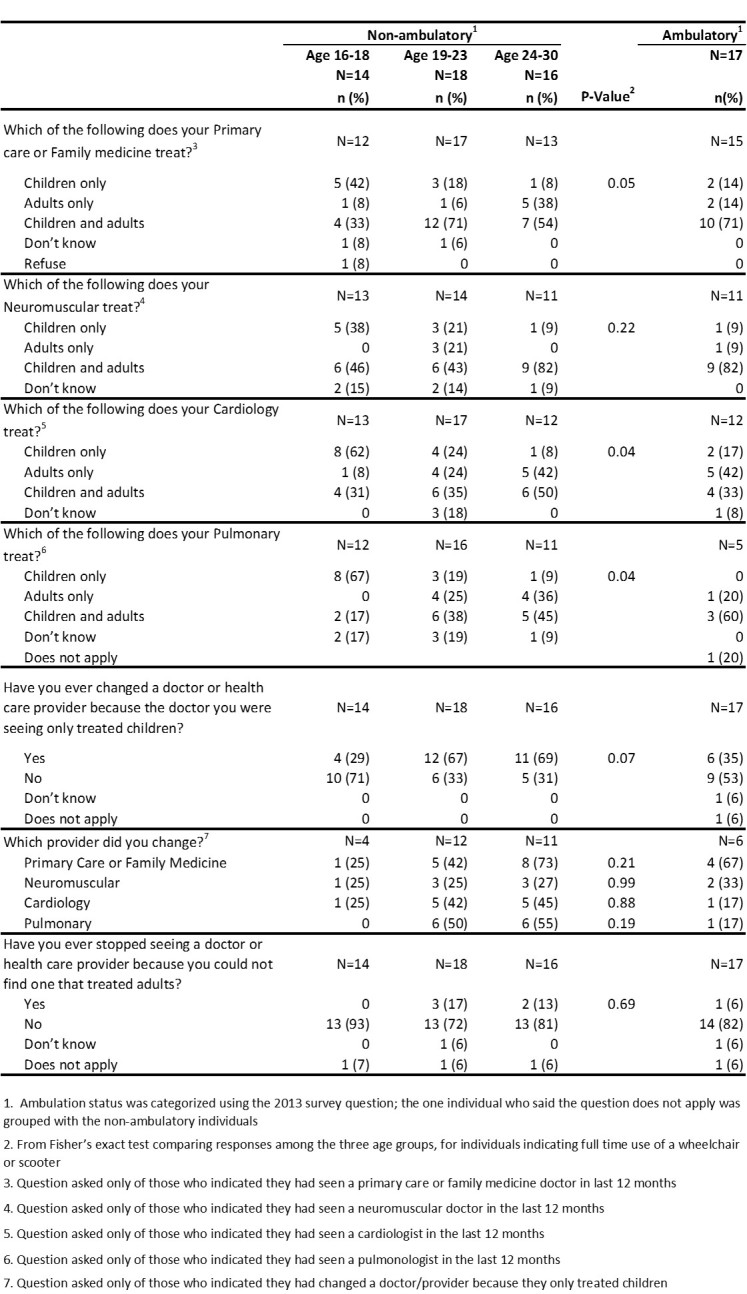
**Table 4.** Types of patients current provider treats and healthcare changes by age and ambulation status, 2013 MD STARnet Health Care Transitions and Other Life Experiences Survey of males affected with Duchenne/Becker muscular dystrophy, aged 16-30 years

Table 5 displays results from questions on discussions about transition health care needs with health care providers and care coordination. Among non-ambulatory males aged 16-18 years, 57% reported talking with their health care providers about eventually seeing different doctors. A high percentage (81-83%) of non-ambulatory males aged 19-30 reported they had help arranging or coordinating their care among different doctors or services. Parents and other family members (63-100%) were the ones who most frequently helped coordinate care, compared to the low percentage of primary care offices (13-22%) and neuromuscular offices (13-44%).

**Figure:**
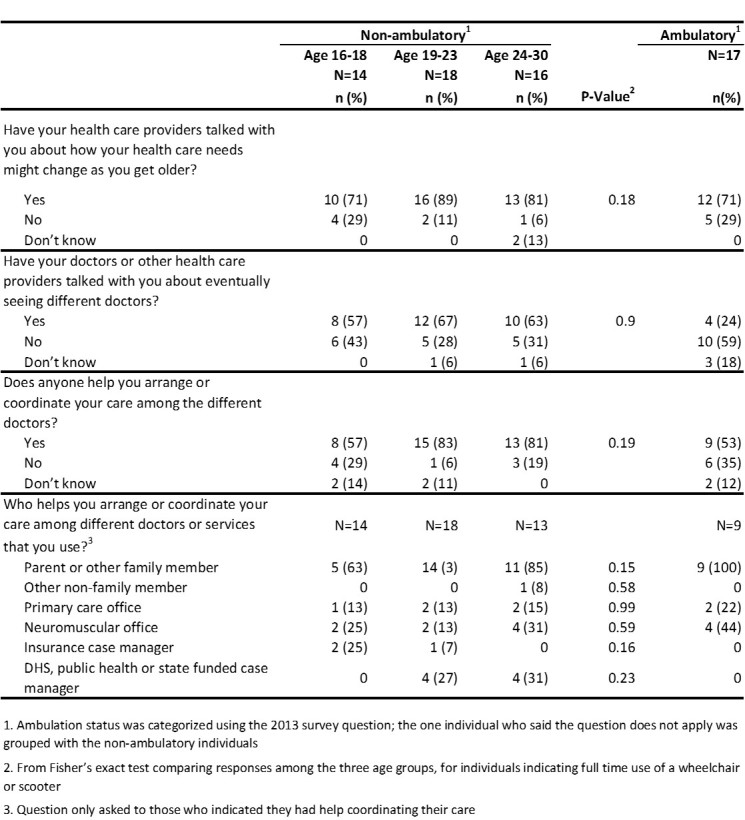
**Table 5.** Health care provider discussions and care coordination by age and ambulation status, 2013 MD STARnet Health Care Transitions and Other Life Experiences Survey of males affected with Duchenne/Becker muscular dystrophy, aged 16-30 years

Table 6 displays results from questions on health care transition planning, insurance, and barriers to care. Among all males, a low percentage (18-28%) reported having a written summary to assist in the transition from a pediatric to an adult health care provider. Among the 15 males who had a written transition plan, 5 (33%) indicated their parent helped develop the plan, while 9 (60%) indicated their pediatrician’s office, family medicine office, or neuromuscular office helped develop the plan; only one male helped develop his plan. Among non-ambulatory males, males aged 16-18 were less likely to have ever had discussions regarding health insurance as compared to older males. The oldest non-ambulatory males were also more likely to report having delayed or gone without needed health care in the past 12 months as compared to the youngest non-ambulatory males (19% vs. 0%).

**Figure:**
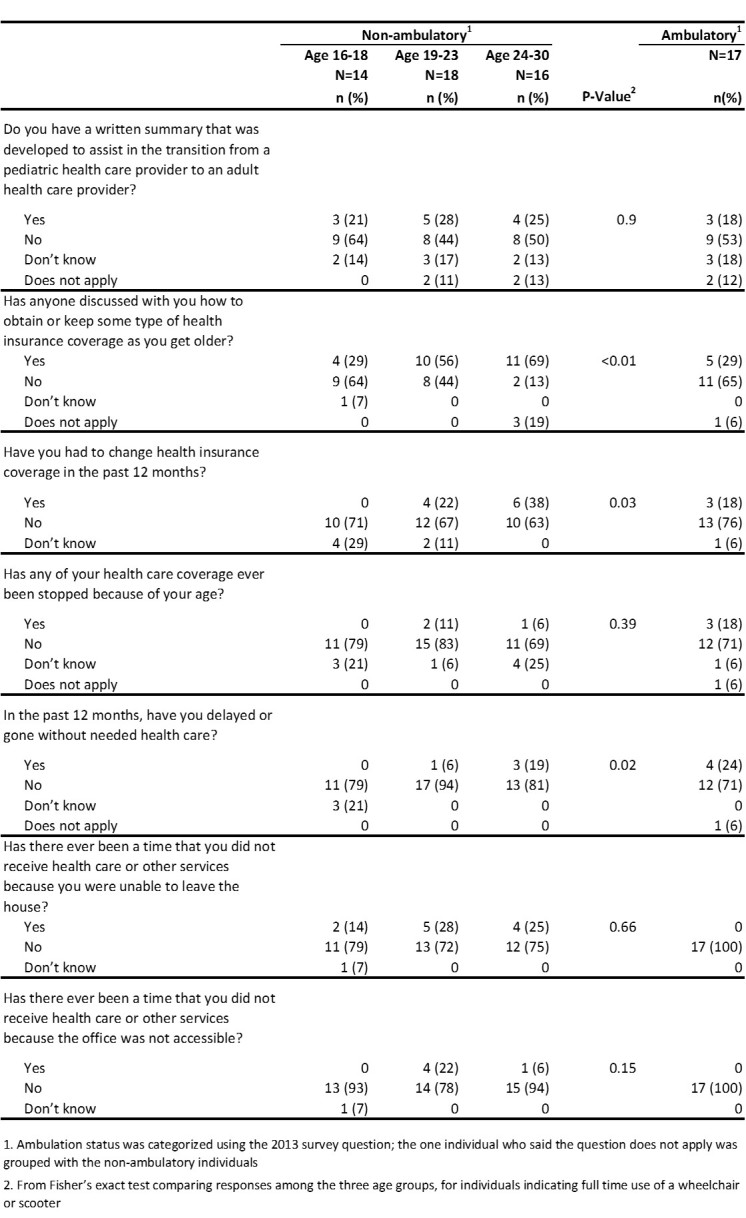
**Table 6.** Health care transition plan, insurance, and barriers to care by age and ambulation status, 2013 MD Health Care Transitions and Other Life Experiences Survey of males affected with Duchenne/Becker muscular dystrophy, aged 16-30 years

## Discussion

The American Academy of Pediatrics, American Academy of Family Physicians, and the American College of Physicians have recommended that formal written transition plans be initiated for all adolescents starting at the age of 14 years.^32^ In this study only 1 in 4 males reported having a written summary to assist in the transition from a pediatric health care provider to an adult health care provider. Approximately 2 out of every 3 males aged 19-30 years reported ever changing a health care provider because their doctor only provided care for children, indicating that the majority of young men with DMD are making a health care transition to adult providers. The lack of written summary may be due to a lack of care coordination services. In this study, approximately 1 in 10 males had a primary care office and fewer than 1 in 3 males had a neuromuscular office help coordinate their care. In a recent national survey of pediatricians, those patients who had a care coordinator were more likely to develop written transition plans.^33^ Care coordination has also been associated with a lower likelihood of having an unmet specialty care need 34 and reduced functional disabilities 35 among children with special health care needs.

A higher percentage of males in our study (71%-89%) reported ever discussing how their health care needs might change as they get older with a health care provider, as compared to the 2007 SATH 29 (55%) and the 2009-2010 NS-CSHCN^28^ (59%) national surveys. Heath care providers of males with DBMD may be more likely to discuss future health care needs than health care providers of patients with other health care conditions.

In our study, 6 of 10 non-ambulatory males reported having ever discussed eventually seeing adult doctors with their pediatric health care providers, which was higher than the NSCSHCN (4 of 10 )^28^ However, for males who were ambulatory in our study, 1 in 4 reported having discussed eventually seeing adult providers with their pediatric health care providers.

In our study, 56-69% of the non-ambulatory males aged 24–30 years reported ever having a discussion with health care providers regarding health insurance as compared to only 29% of non-ambulatory males aged 16–18 years. The percent of participants who reported discussing health insurance with their providers was 35% in the NS-CSHCN^28^ and 53% in the SATH^29^. It is important that health insurance discussions occur before a transfer to adult health services. Approximately half of young adults with disabilities and those with special health care needs have been shown to experience gaps with insurance coverage.^36^^, ^37^, ^38 Young adults with no health insurance are more likely to experience a delayed transition of care than young adults with health insurance.^39^ Young adults with special health care needs who are uninsured have been shown to delay or forgo care and to have problems getting care.^40^ In our study, 4 in 10 non-ambulatory males aged 24-30 reported changing health insurance coverage in the past 12 months as compared to none of the non-ambulatory males aged 16-18 years. The reasons for the high proportion of non-ambulatory males aged 24–30 years who changed their health insurance coverage are unknown; possible explanations include men changing from their parents’ insurance to their own insurance, or qualifying for Medicare or Medicaid.

Lack of health care access may have been an issue for some of the non-ambulatory males aged 24–30 years in our study, with 2 of 10 males delaying or going without needed health care in the past 12 months. One reason for this barrier to care may be a lack of transportation. One in 4 males aged 19–30 years did not receive care because they were unable to leave the home.

The recent increase in survival among males with DMD has been attributed to advances in cardio-pulmonary therapies, including non-invasive ventilation and cough assistance.^41^^, ^42^, ^43^, ^44^, ^45 Among the non-ambulatory males aged 24–30 years, several reported not having a cardiologist or pulmonologist involved in their care in the past 12 months. A transition study of males with DMD living in England obtained similar results: 47% of parents reported their son had contact with a cardiac clinic and 39% of parents reported their son had contact with a respiratory clinic in the last six months.^18^ The 2010 Duchenne muscular dystrophy care considerations state that non-ambulatory males with DMD should have pulmonary assessments at least every 6 months and males with DMD over the age of 10 years should have cardiac assessments at least every year.^46^

Our study has several strengths. It is one of the largest studies in the United States to describe the transition experiences of males living with DBMD. Eligible participants were drawn from a surveillance system that identifies all individuals living with DBMD within specific geographic areas. Our study surveyed the males themselves, while many studies that examine the transition experiences among adolescents and young adults rely on responses from caregivers.

Our study also has some limitations. Whereas our study population was drawn from a population-based surveillance system, we had a survey completion rate of 25% and a small sample size. Males who completed the survey differed from males who did not complete the survey by both state of residence and race/ethnicity, and therefore the experiences of the participants may not reflect the experiences of all males with DBMD in the respective surveillance areas. Also, the results from this survey may not be generalized to all males living with DBMD in the United States. While no differences in functional status were observed at the end of the surveillance period between males who completed the survey and males who did not, it is possible that, differences in functional status may have existed two years later at the time of the transition survey. In our survey, we did not ask the males if a caregiver helped them complete the survey, which may have biased the responses.

Our study found that many adolescent and young adult males living with DBMD lack a written transition plan, and among those who did have a plan, only one male reported participating in its development. The American Academy of Pediatrics, American Academy of Family Physicians, and American College of Physicians-American Society of Internal Medicine recommends that health care providers, parents, and adolescents/young adults jointly participate in the development of transition plans.^1^^, ^32 The results of this study may indicate a lack of targeted informational resources and education focused on supporting the transition of young men with DBMD as they age from adolescence into adulthood within the healthcare system. Recently, updates to the 2010 DMD care considerations were published, including a new section devoted to healthcare transition planning.^7^ Future studies could try to determine the reasons for the potential barriers to health care described in this study as well as identify the optimal transition programs for adolescent and young adult males living with DBMD. Online resources are available to adolescents and young adults with special health care needs, their families, and health care providers, including Got Transition, a program of the national Alliance to Advance Adolescent Health which is supported through a cooperative agreement with the Maternal and Child Health Bureau of the Health Resources and Services Administration.^47^ There is also an online resource specific to adolescents and young adults with neuromuscular disorders, the Young Adult Programs from the MDA.^30^

## Corresponding Author

Pangaja Paramsothy

Mailing Address: Division of Human Development and Disability, National Center on Birth Defects and Developmental Disabilities, Centers for Disease Control and Prevention, MS E88, 4770 Buford Hwy., Atlanta, GA 30341

Email: pparamsothy@cdc.gov

## **Data Availability**

Due to privacy concerns (detailed personal information was obtained from a small number of individuals living in a defined surveillance area), data from MD STARnet Health Care Transitions and Other Life Experiences Survey is not publicly available. We are willing to work with individuals who would like to verify the results.

Data from this analysis is kept at the Centers for Disease Control and Prevention. Researchers interested in utilizing MD STARnet data should contact: Natalie Street at the Division of Human Development and Disability, National Center on Birth Defects and Developmental Disabilities, Centers for Disease Control and Prevention, Mailing Address: Division of Human Development and Disability, National Center on Birth Defects and Developmental Disabilities, Centers for Disease Control and Prevention, MS E88, 4770 Buford Hwy., Atlanta, GA 30341. Email: nstreet@cdc.gov

## Competing Interests

The authors have declared that no competing interests exist**.**
